# Detection of Bocavirus in Children Suffering from Acute Respiratory Tract Infections in Saudi Arabia

**DOI:** 10.1371/journal.pone.0055500

**Published:** 2013-01-30

**Authors:** Ahmed S. Abdel-Moneim, Mahmoud M. Kamel, Abdullhamid S. Al-Ghamdi, Mater I. R. Al-Malky

**Affiliations:** 1 College of Medicine, Taif University, Al-Taif, Saudi-Arabia; 2 Department of Virology, Faculty of Veterinary Medicine, Beni-Suef University, Beni-Suef, Egypt; 3 Department of Clinical Pathology, National Cancer Institute, Cairo University, Cairo, Egypt; 4 Pediatric Hospital, Al-Taif, Saudi Arabia; University of Kansas Medical Center, United States of America

## Abstract

Human bocavirus (HBoV) was recently discovered in children with respiratory distress and/or diarrhea. To our knowledge, no previous study has reported the existence of bocavirus in Saudi Arabia. Swabs samples from 80 children with respiratory tract infections were examined for the presence of HBoV. Real-time polymerase chain reaction was used as a sensitive method to detect the HBoV. Direct gene sequencing was used to determine the genotype of the detected virus isolates. HBoV was detected in 22.5% of the examined patients. The NP1 partial gene sequence from all patients showed that the circulated strains were related to HBoV-1 genotype. Most of HBoV infected patients showed evidence of mixed coinfection with other viral pathogens. The current study clearly demonstrated that genetically conserved HBoV1 circulates in Saudi Arabia. Interestingly, most of the HBoV1 infected cases were associated with high rates of co-infections with other viruses.

## Introduction

Bocavirus is a single-stranded DNA virus belonging to the family *Parvoviridae*, subfamily *Parvovirinae*, genus *Bocavirus*. Bocaviruses are unique among parvoviruses because they contain a third ORF between the non-structural and structural coding regions [1]–[2]. The genus bocavirus includes viruses that infect bovine, canine, feline, porcine and some simian species as well as sea lions [3]–[8]. Human bocavirus (HBoV) was first found in children with acute respiratory tract infections in 2005 [1]. It was then detected in children with respiratory tract infections in addition to gastroenteritis worldwide [9]–[12]. The virus exists in four different serotypes HBoV1-4 [1]–[2], [13]–[14]. Although HBoV 1 and 2 were reported in respiratory samples, all the 4 genotypes of HBoV have been identified in children with acute gastroenteritis.

HBoV has been reported in various countries, indicating its worldwide endemic nature. The virus has been identified in Europe [15]–[17], America [18]–[19], Asia [9], [20], Australia [21]–[22], Africa [23], and the Middle East [24]. The prevalence of HBoV ranges between 1.5 to 19.3% [18], [25]. Primary infection with HBoV seems to occur early in life and children between the ages of 6–24 months seem to be mostly affected [9]–[10], but older children can also be infected. Newborn children may become protected by maternally derived antibodies [9]. HBoV infections are rarely found in adults [26]–[27]. Lindner et al. detected anti-HBoV antibodies in 94% of healthy blood donors >19 years of age [28].

HBoV detection has been mostly performed on nasopharyngeal aspirates and swabs and relies mostly on classical [10], [18], [19], [21], [22], [26] and real-time PCR [10], [23], [29]. Real-time PCR possesses many advantages over conventional PCR, as it offers greater sensitivity, specificity, and reduced expenditure of time.

The current study aims to screen the epidemiological status and molecular phylogeny of HBoV isolates prevailing in pediatric patients with respiratory infection in Saudi Arabia.

## Results and Discussion

The current study investigated the prevalence of HBoV in patients suffering from respiratory tract infections in Saudi Arabia. The presence of the major viral causes of the respiratory distress in HBoV positive cases was also screened. HBoV was detected in 18/80 of the examined patients (22.5%) with ages ranging from 2 months to 10 years, (Table 1–2). Clinical findings for HBoV-positive patients were indistinguishable from those for patients with other respiratory viruses. Previously, HBoV has been detected in samples from patients aged between 5 months and 2 years [1], [29]. Ma et al, speculated that the antibody against HBoV derived from the mother might protect children under 5 months of age from HBoV infection [9], however, we detected HBoV in two cases below 5 months: in a 2-month-old and 4-month-old child (Table 1–2) that may indicate the possibility of HBoV infection in very young children.

**Table 1:** Demographic and clinical data of children suffered from respiratory distress in the current study.

Variable	Value [N = 80]	HBoV infected [N = 18]
Age	2 mo- 10 yr	
0–4 Mo	11	2
5–8 Mo	6	1
9–12 Mo	1	1
1–2 yr	27	8
>2–3 yr	8	4
>3–4 yr	4	0
>4–5 yr	6	1
>5–7 yr	4	0
>7–10 yr	4	1
Sex		
Male	39	8
Female	41	10
Clinical symptoms		
Fever	80	18
Nasal discharge	80	18
Watery	70	16
Purulent	10	2
Asthma	16	2
LRTI^a^	41	15
URTI^b^	39	3
Diarrhea	20	3
Nervous manifestations	2	0
Rash	6	0

a Lower respiratory tract infection.

b Upper respiratory tract infection.

**Table 2:** Common respiratory viruses among HBoV infected children.

Sample No	Age	Sex	RSV	PIV-1	PIV-3	IAV	Adenovirus
1	2 Mo	Female	+	−	−	−	−
2	10 Mo	Male	−	−	−	−	−
3	8 Mo	Male	−	−	−	+	−
4	18 Mo	Male	+	−	−	+	−
5	19 Mo	Male	+	−	−	−	−
6	36 Mo	Female	+	−	+	+	−
7	12 Mo	Female	+	−	−	+	+
8	36 Mo	Male	+	−	−	−	−
9	17 Mo	Female	+	−	−	+	+
10	13 Mo	Female	+	−	−	+	+
11	5 yr	Female	+	−	−	+	+
12	4 Mo	Female	+	−	−	+	+
13	20 Mo	Male	+	−	−	+	−
14	20 Mo	Male	−	−	−	+	−
15	24 Mo	Female	−	−	−	+	+
16	10 yr	Female	−	−	−	+	−
17	30 Mo	Female	+	−	−	−	−
18	32 Mo	Male	+	−	−	−	−

The rate of HBoV in respiratory tract infections has been reported to be 1.5 to 19.3% [18], [25]. Real-time PCR was used in the current study to screen HBoV due to its high diagnostic sensitivity that could be responsible for the higher rate of HBoV infection in Saudi Arabia than the widely accepted upper limit of infection rates worldwide. Meanwhile, a recent study showed 21.5% prevalence among children [30].

The evidence of HBoV as the main initiator of the disease in the infected cases is still uncertain because of its high co-infection rate with other pathogens, and it remains unclear whether HBoV is the sole etiologic agent or just a concomitant virus bystander. In previous studies, none of the nasal swabs obtained from healthy children yielded a positive HBoV test. This suggests that HBoV is not a frequent commensal virus inhabiting the respiratory tract [16], [19]. HBoV infections are frequently present in concomitant with other viruses and often occur in more than 50% of the tested samples [31]. In the current study, only one case was found to be infected only by HBoV as a single virus entity while most of isolates (17/18) showed coinfection with other viral pathogens. The most frequently detected co-pathogens were RSV (13/18; 72.2%), IAV (12/18 cases, 66.66%), respiratory adenovirus (6/18 cases, 33.33%) while only 1/18 (5.5%) case was coinfected with PIV-3 and none was coninfected with PIV-1 (Table 2). It is assumed that the rate and frequency of coinfections may be higher if more viruses were screened. Consistent with other studies [1], [16], the prevalence rate of bocavirus was higher in children under 2 years of age (Table 1).

**Figure 1:**
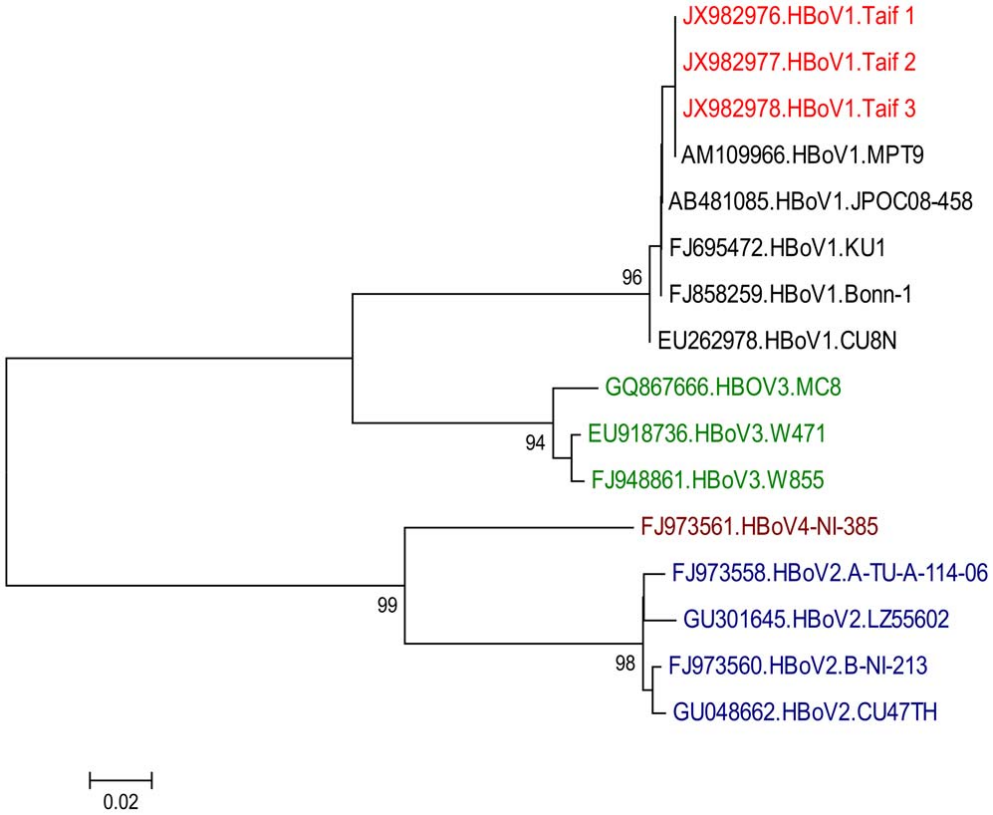
Phylogenetic analysis of Saudi HBoV1 isolates. The phylogenetic tree with 1,000 bootstrap replicates was generated using the Clustal W program in the MEGA 4.0 software package and based on partial NP-1 sequences of the HBoV strains. Samples obtained in the current study from children with acute respiratory distress in Saudi Arabia are in red. HBoV1 strains are presented in black, HBoV 2 strains are presented in blue, HBoV3 strains are presented in green while HBoV4 isolate is presented in brown.

Partial NP-1 gene sequence of the eighteen detected HBoV strains were obtained in our study. Multisequence analysis showed complete identity (100%) between each other, and phylogenetic analysis demonstrated that they belonged to HBoV1 (data not shown). Blast analysis revealed complete homology to the published sequence of HBoV1. Furthermore, the phylogenetic analysis results of three selected sequences showed that the Saudi HBoV1 strains obtained from respiratory samples belonged to group I human bocaviruses (Fig. 1).

To the best of our knowledge, this is the first report of HBoV1 in Saudi Arabia. Continuous surveillance and genome sequence analysis are needed to obtain more information on the genotypic variation and molecular evolution of HBoV in the country.

## Materials and Methods

### Ethics Statement

The study protocol was approved by the medical ethics review board of the College of Medicine, Taif University and by the pediatric hospital ethics committee in accordance with the guidelines for the protection of human subjects. Informed written consents from the next of kin of the participants involved in the study were taken.

### Sample Collection and Processing

Nasopharyngeal swabs from 80 children suffered from moderate to severe lower respiratory tract infections were collected from January to May 2012 from the Governmental Pediatric Hospital- Al-Taif, Saudi Arabia. The children’s age ranged from two months to ten years of age. Clinical manifestations and case histories were recorded. Individual swabs were kept in 1 ml sterile saline containing gentamycin sulphate. Swabs were routinely processed and kept at −80°C until further analysis.

### Nucleic Acid Extraction

Viral nucleic acid was extracted from 200 µl of individual samples using DNA/RNA extraction Kit (Koma Bioteck Inc., Seoul, Korea), according to the manufacturer’s instructions.

### Real Time Polymerase Chain Reaction (RT PCR)

The real-time PCR assay was performed using commercial, TaqMan hydrolysis probe based, real time PCR bocavirus detection Kit (Liferiver, Shanghai, China) in Eppendorf Mastercycler® ep realplex^2^. The detection of the amplified amplicon was performed in fluorimeter channel FAM with the fluorescent quencher BHQ1. Amplification reactions were performed in a volume of 25 µl containing 2.5 µl of DNA template, 21.5 µl reaction mix, 0.4 µl enzyme mix, 1 µl internal control according to the manufacturer’s instructions. The thermal cycling conditions were as follows: 2 min at 37°C, an initial denaturation of 2 min at 94°C and 40-cycles of 15 sec at 93°C and annealing/elongation step of 1 min at 60°C. HBoV positive samples were screened for the presence of respiratory syncytial virus (RSV), influenza A virus (IAV), parinfluenza virus 1(PIV-1) and parainfluenza virus 3(PIV-3), as well as respiratory enteric virus (AdenV) using real-time PCR Kits (Shanghai ZJ Bio-tech Co., Ltd).

### HBoV Genotyping

PCR amplification of a 354–base pair fragment of the NP1 was performed as described previously [1]. The reaction mix contained 20 pmol of each primer and DNA master mix (Koma Bioteck, Inc., Seoul, Korea). The thermal cycling conditions were as follows: an initial denaturation of 5 min at 94°*C*, 35 cycles of 1 min at 94°C, 1 min at 54°C and 2 min at 72°C, final extension of 10 min at 72°C.Positive PCR products were purified using a QIAquick PCR purification kit (Qiagen) and were sequenced commercially (Macrogen Inc., Seoul, Korea).

### Sequence Analysis

The nucleotide sequences of the NS1 gene were compared with those of HBoV strains available at the GenBank site. Phylogenetic analyses were conducted with MEGA, version 4.1. The 3/18 partial sequences of the NS1 gene were submitted to GenBank (accession numbers JX982976–JX982978).
